# Temporal and intra-horse consistency of circulating myostatin concentrations in Thoroughbred racehorses

**DOI:** 10.1038/s41598-025-22472-7

**Published:** 2025-11-05

**Authors:** Katherine Hanousek, Victoria O’Hara, Dominique O. Riddell, Richard J. Piercy

**Affiliations:** Comparative Neuromuscular Diseases Laboratory, Royal Veterinary College, London, UK

**Keywords:** Genetics, Physiology

## Abstract

In Thoroughbred horses, a highly prevalent short interspersed nuclear element (SINE) mutation in the myostatin gene (*MSTN*) promoter influences circulating myostatin concentration and is associated with muscle morphology, fracture risk and optimal race distance. Our prior data reveal that within horses there is substantial variation in serum myostatin concentration, particularly in *MSTN* heterozygotes and wild type horses but it was unclear whether such variation relates to within-horse differences or to environmental or temporal effects. Here we report the intra-horse consistency of circulating myostatin, and investigated the hypothesis that it is affected by season. 49 racing Thoroughbreds under identical management were genotyped for the *MSTN* promoter SINE insertion and myostatin concentrations were measured in plasma with a validated enzyme-linked immunosorbent assay, from blood samples collected repeatedly over a year. As expected, genotype was significantly associated with circulating myostatin, with homozygotes having the lowest concentration, followed by heterozygous and then wild-type horses. Intra-horse plasma myostatin had low to moderate variability throughout the year, within genotyped groups (coefficient of variation: 17 to 23%): aside from a direct mutation effect, horses tended to be low, moderate, or higher myostatin expressers. Neither season, sex, nor age significantly influenced plasma myostatin concentration. Further work is necessary to determine whether an individual’s plasma myostatin concentration is associated with any performance advantages, muscle characteristics or fracture risk within SINE genotyped groups.

**Supplementary Information:**

The online version contains supplementary material available at 10.1038/s41598-025-22472-7.

## Introduction

Myostatin, (growth/differentiation factor-8) expressed by skeletal muscle, acts as a negative regulator of skeletal muscle growth^1–4^. The myostatin gene (*MSTN*) is well conserved across mammalian species: null mutations have been identified in cattle^5^, and dogs^6^, and other variants influence muscle conformation in pigs^7^ and sheep^8^. Studies in *MSTN* null mice have demonstrated increased muscle mass associated with both fibre hypertrophy and hyperplasia compared to wild-type littermates^1^. Further, *MSTN* null mice have more glycolytic and fewer oxidative muscle fibres^9–11^. Myostatin’s role in muscle is (likely indirectly) also associated with compensatory alterations in bone mass: it thus has an important role in both bone formation and regeneration^12,13^. Inhibition of myostatin in early bone healing enhances repair via increases in callus size and bone volume^14^.

*MSTN* variants, and specifically an intron 1 single nucleotide polymorphism (SNP) are linked to optimum race distance^15,16^ and body morphology^17^ in Thoroughbreds, and are selected for in Quarter Horses^18^. Additionally, a short interspersed nuclear element (SINE) mutation is present in the *MSTN* promoter^19,20^, in Thoroughbreds and Quarter Horses. A commercially available genetic test for the SNP is advocated for use in Thoroughbred racing and training to determine race distance aptitude^21^. However, the linked SINE is the functional mutation (linkage disequilibrium exists between the two mutations^18^); the SINE mutation resulted in a 4.5 fold lower myostatin protein production in vitro*,* while the SNP mutation alone had no effect^22^. In vivo, the SINE mutation is associated with low circulating (plasma) myostatin: the magnitude of the reduction inversely associated with the number of mutant alleles (i.e. homozygotes < heterozygotes < wild type horses)^23^. Similar to null rodent models, equine homozygotes with the *MSTN* SINE have a higher proportion of 2X muscle fibres and fewer type 1 fibres than wild type horses^19^. Furthermore, the linked intron 1 SNP is associated with susceptibility to carpal fracture in Thoroughbred racing, perhaps because of enhanced speeds and higher bodyweight^24^.

While the SINE mutation significantly impacts circulating myostatin concentrations in Thoroughbred horses, we have shown that there is notable overlap between genotyped groups, particularly within wild type horses and heterozygotes^23^. This suggests that other genetic or environmental factors might influence circulating myostatin in horses, feasibly also then influencing muscle mass, oxidative capacity and bone health. In racing Thoroughbreds, plasma myostatin was not associated with sex or age^23^; however higher myostatin expression was demonstrated 30 minutes following a single bout of exercise in Arabian horses, though not by 4 months of training^25^. Circulating myostatin is also associated with obesity^26^, though this is unlikely to be relevant in most racehorses.

Seasonal variation in plasma myostatin occurs in house sparrows^27^ and Djungarian hamsters^28^; sparrows experience a drop in myostatin with an associated increased muscle mass over winter in cold climates, while hamsters have increased myostatin over winter (regulated by day length). Seasonal variation of myostatin expression also occurs in humans with a peak seen in the spring^29^. Individual and seasonal variation have not been described in horses, which are known otherwise to be hormonally influenced by photoperiod^30^.

Improved understanding of differences in myostatin expression within and between horses might help further explain variations in muscle mass, exercise physiology and bone health, with possible welfare and financial implications in racing. Consequently, the objectives of this study were to assess the hypotheses that there is seasonal variation in myostatin expression in horses and consistency of myostatin expression within individual horses of defined *MSTN* genotypes.

## Methods

### Ethics

The study was approved by the Clinical Research Ethical Review Board of the Royal Veterinary College (URN: 2023 2195-2) and was conducted in accordance with all relevant guidelines and regulations. Owner consent was obtained.

### ARRIVE statement

Experimental animals were not used for this study however, where appropriate, methods, statistical analysis and experimental design were conducted according to ARRIVE 2.0 guidelines as noted.

### Sample collection.

Residual EDTA and clotted blood samples were obtained from 49 Thoroughbred racehorses from one flat racing training yard in England, at approximately monthly intervals between April 2023 and April 2024. Samples were obtained in the afternoon (3–3:30 pm) following light training exercise in the morning (7–8:30am, typically light exercise performed under saddle on gallops). All horses were housed in single, outdoor stables bedded on wood shavings and fed a diet of dry hay and racehorse cubes. All horses were in flat race training at the time of sampling. Age, sex, date of foaling and year of foaling were recorded for all horses. Months were apportioned to season, with March to May considered spring, June to August, summer, September to November, autumn, and December to February, winter.

### Genotyping

A single residual aliquot of whole EDTA blood from each horse was stored at − 80 °C within 72 h. Subsequently, DNA was extracted using a commercial kit, as per manufacturer’s instructions (Illustra Nucleon BACC 3 Genomic DNA extraction kit, G E Healthcare) and as previously described^23^. Briefly, concentration and purity were measured (NanoDrop One; ThermoFisher Scientific) and polymerase chain reaction (PCR) was used to determine the presence of the 227 base pair SINE insertion in the *MSTN* promotor, using AmpliTaq Gold (Invitrogen) and associated reagents and the following primers: 5′–CTG ACA TTA TGC CCT GGT AA–3′ (Forward), 5′–CGC TGT TCT CAT TTA GAT CC–3′ (Reverse). The SINE mutation in homozygotes was revealed by a PCR product of 1210 bp, whereas wild type horses had a product of 983 bp. Heterozygous horses had both bands.

### Quantifying plasma myostatin.

Myostatin was quantified by ELISA (enzyme-linked immunosorbent assay, GDF-8/Myostatin ELISA: R&D systems) according to manufacturer’s instructions as previously described^23^. Samples were diluted 1:10 with Calibrator Diluent RD5-26 and optical density was measured at 450 nm with wavelength correction at 570 nm. Controls of sterile water and a buffer solution were used for every plate (not shown). To examine the impact of both matrix and storage on stability, myostatin concentrations were measured in serum and plasma samples in parallel from five horses with samples separated and frozen at − 80 °C immediately after sampling, after 24 h storage at room temperature, after 24 h storage at 5 °C, and after 72 h at 5 °C. For remaining analyses, plasma was separated and frozen at − 80 °C within 72 h from samples previously stored at 5 °C. Inter- and intra-assay coefficient of variation (CV %) was calculated from duplicate samples and repeated samples across plates.

Myostatin concentration was measured in technical duplicates in all plasma samples obtained from each horse over the year. The intra-horse CV % of plasma myostatin concentration was calculated for each animal for which 3 or more plasma samples at different dates were available.The inter-horse CV% of plasma myostatin within each genotype was calculated from the first sample obtained from each individual. In order to examine the possible influence of day to day haemodynamic changes on plasma myostatin concentration (perhaps related to recent exercise or dehydration), we additionally measured plasma albumin in the three horses per genotype (n = 9) with the highest CV % to calculate a myostatin: albumin ratio CV %, and compared this with the raw myostatin CV % for each animal.

### Statistical analysis

Data were analysed in R (R Core Team, 2022, Vienna, Austria). The code and full model outputs are provided in Supplementary File 1. Shapiro–Wilk test for normality and Levene’s test for homogeneity of variance were applied performed to guide choice of statistical tests. Influence of storage and matrix (serum vs. plasma) were examined by two-way repeated measures analysis of variance (ANOVA). Comparisons of myostatin concentrations between genotyped groups were conducted on the mean myostatin concentrations measured for each animal over the year, by ANOVA (with Welch’s correction to account for unequal variances). Linear mixed effects models were constructed using the lmerTest package. Genotype, season sampled, sex, age and their interactions were included as fixed effects. Horse identity and ELISA plate were included as random effects. Normality of the data was visually assessed using histograms of the residuals, and significance of main effects and interactions were assessed with type III ANOVA. A paired Student’s t-test with Bonferonni correction was used to assess whether accounting for haemoconcentration had a significant impact on myostatin variance. Proportions of genotyped horses sampled in different seasons were compared by Chi-squared test. Differences or associations were considered statistically significant when p < 0.05.

## Results

### Population

Repeated blood samples were collected over the course of a year from 49 horses that comprised 27 geldings, 16 mares and 6 stallions, with a median (IQR) age of 3.5 (1.5) years at time of initial blood sampling. Nine horses were *MSTN* SINE homozygotes (1 mare, 6 geldings, 3 stallions), 23 were heterozygotes (11 mares, 11 geldings, 1 stallion) and 17 were wild type (4 mares, 11 geldings, 3 stallions). The median number of blood samples obtained over the study duration per horse was 5 (IQR 2) for heterozygous horses, 5 (IQR 3) for homozygous horses, and 6 (IQR 2) for wild type horses. There was no significant difference in the distribution of each genotype across seasons (p = 0.33). The intra-assay CV% was 4.0% and the inter-assay CV% was 8.5%.

### Sample stability and matrix

No statistically significant differences were found in myostatin concentration measured in plasma or serum under any of the tested storage conditions (p = 0.67), or between serum and plasma (p = 0.54; data not shown).

### Genotype

**Fig. 1:**
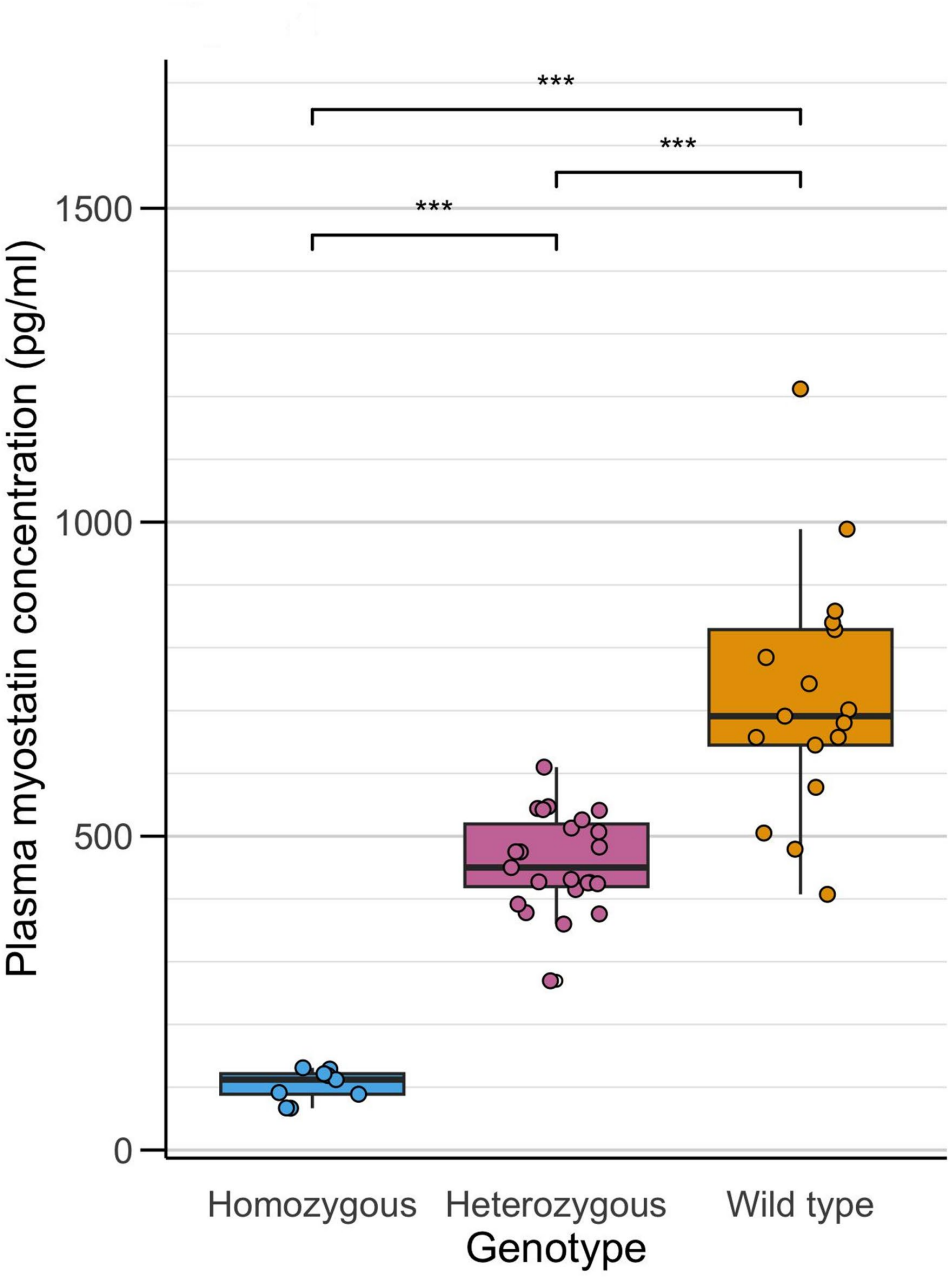
Myostatin concentration across genotypes. Boxplot showing plasma myostatin concentration in horses of each genotype. Each data point represents the mean myostatin concentration for an individual horse obtained from all samples over the year. A significant difference is observed between all genotypes (***p < 0.001).

As expected, genotype had a highly significant impact on myostatin concentrations (Fig. 1): homozygotes had lower mean plasma myostatin concentration than heterozygous horses (p < 0.001), and heterozygous horses had lower mean myostatin concentration than wild type horses (p < 0.001). Plasma myostatin concentrations varied within genotyped groups and there was prominent overlap between those measured in heterozygotes and wild type horses (Fig. 1). Across all genotypes, the range of plasma myostatin seen was 38.35 pg/ml to 1472.95 pg/ml. There was no significant difference in seasonal distribution of genotypes (p = 0.33).

### Mixed model results

**Fig. 2:**
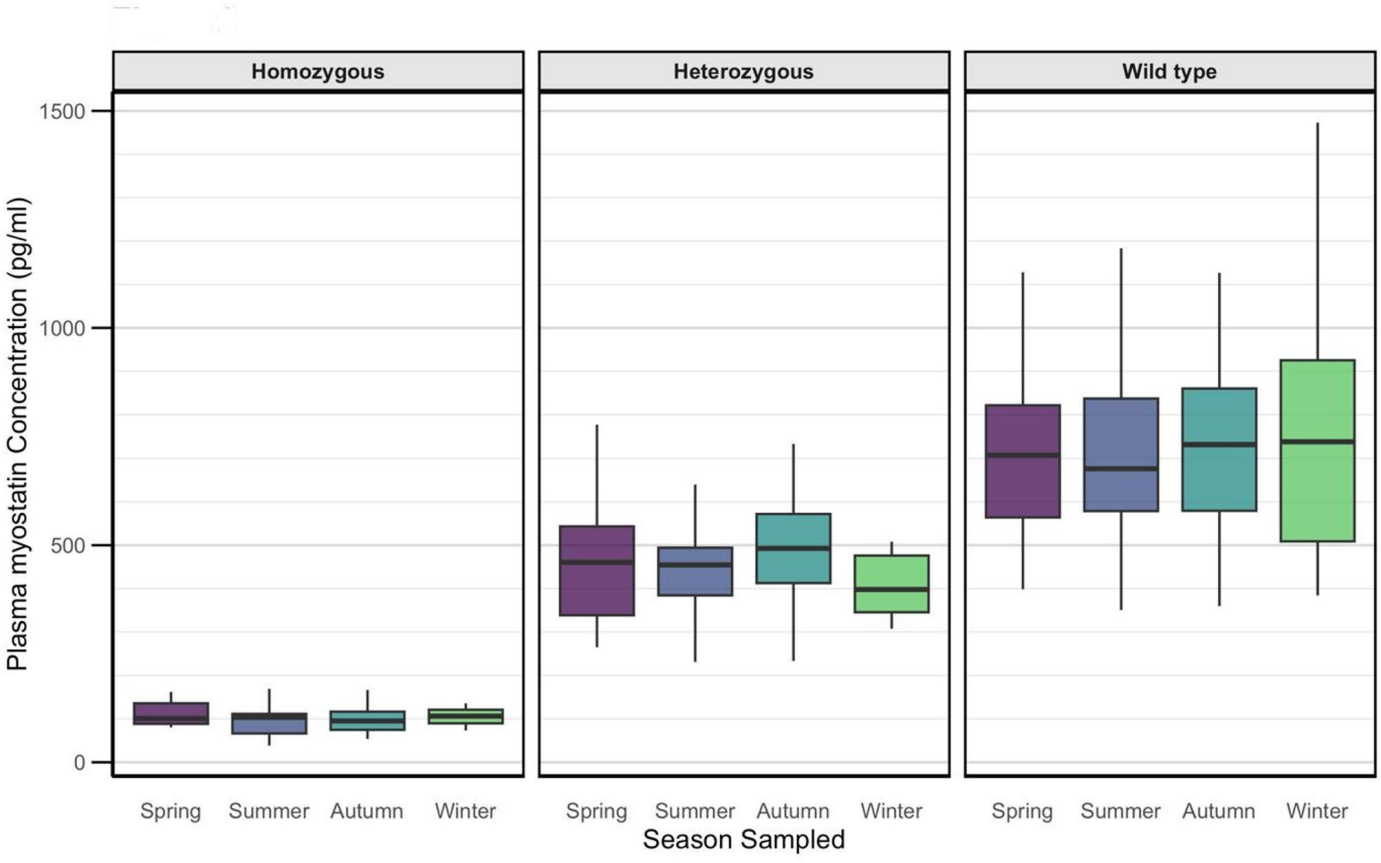
Seasonal variation in myostatin concentration by genotype. Boxplot showing plasma myostatin concentrations measured in each season, separated by genotype. No significant differences were observed between seasons within each genotype (p = 0.08).

**Fig. 3:**
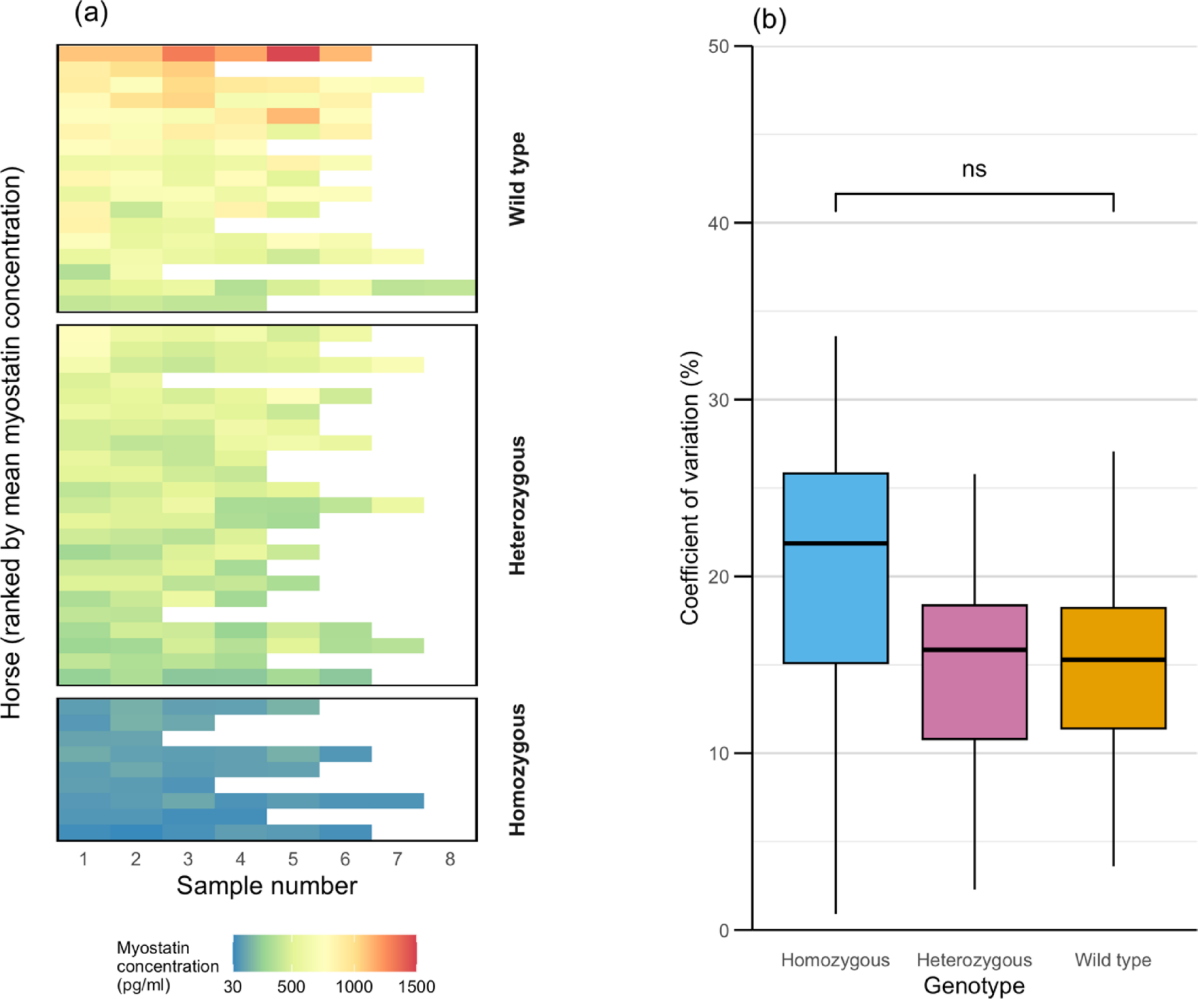
Individual and within-horse variation in myostatin concentration. (**A**) Heatmap showing myostatin concentrations across sequential samples (in chronological order, from left to right) for individual horses, ranked by mean myostatin value for each individual (top to bottom). (**B**) Boxplot displaying the coefficients of variation (%) for horses contributing more than three samples, separated by genotype. No significant difference in coefficient of variation was observed between genotypes (ns, p = 0.21).

There were no significant differences between plasma myostatin in different seasons sampled (p = 0.08; Fig. 2); further, there was no significant interaction between these variables and genotype (p = 0.77). In general, plasma myostatin remained relatively consistent within individual horses (Fig. 3A) There were no differences in plasma myostatin concentration between sexes (p = 0.31) or in horses of different ages (p = 0.71), and no interaction between sex or age and genotype (p = 0.73, p = 0.98, respectively).

### Coefficient of variation

**Fig. 4:**
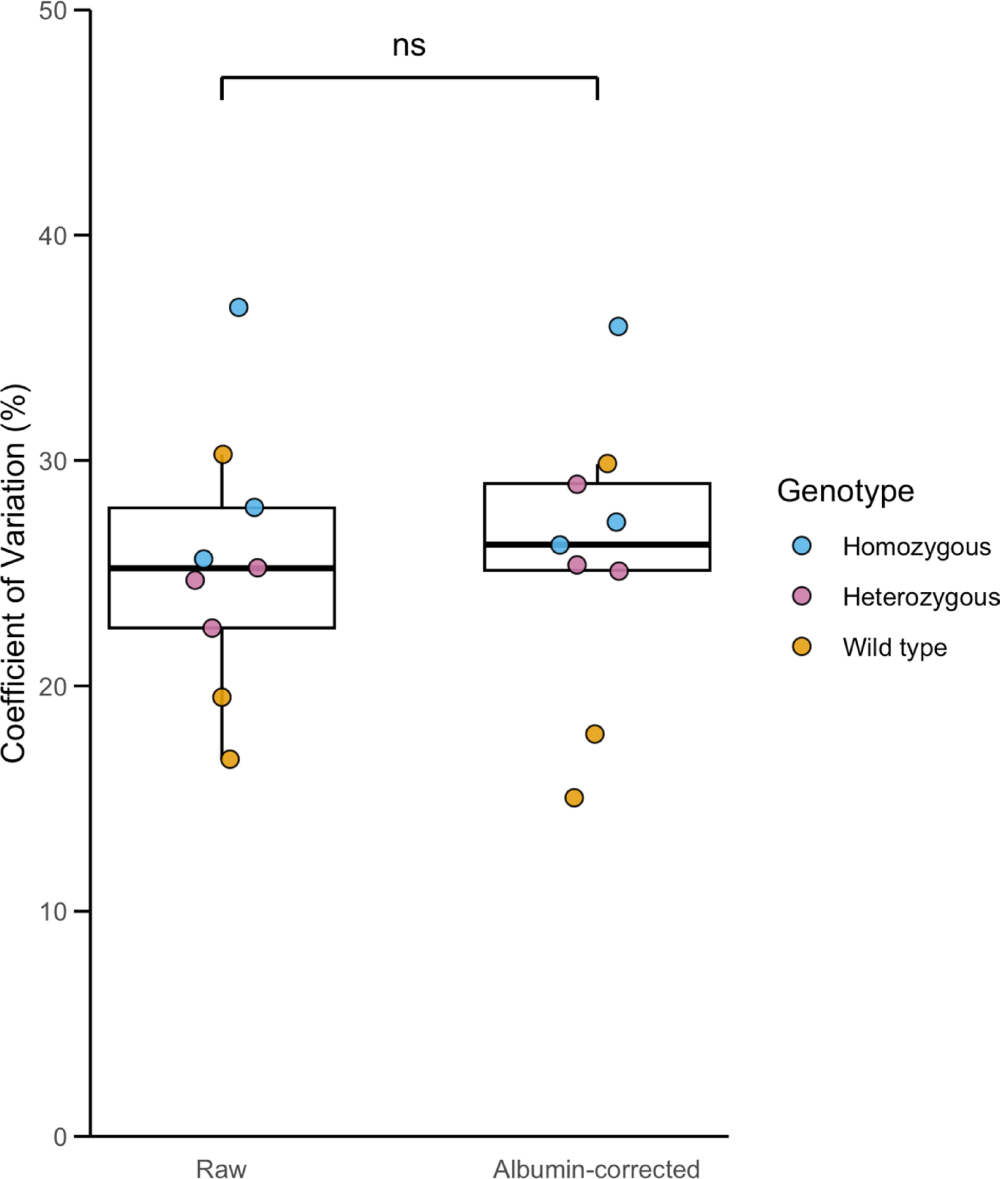
Coefficient of variation in raw and albumin-normalised myostatin. Box plot showing the coefficient of variation (%) for raw myostatin and albumin-normalised myostatin, with individual horses (n = 9) coloured by genotype. No significant differences were observed in the coefficient of variation for either measure when comparing genotypes (ns, p = 0.71).

There was no significant difference in CV % of plasma myostatin between the genotypes (p = 0.21): the mean intra-horse CV % for plasma myostatin was 17% (SD = 6%) for heterozygous horses, 18% (SD = 9%) for wild-type horses and 23% (SD = 10%) for homozygotes (Fig. 3B). There was no difference in the CV % when myostatin data were normalised to albumin concentration (26%, SD = 6%) compared with the raw values (25%, SD = 6%, p = 0.71; Fig. 4). The inter-horse CV % for heterozygous and homozygous horses was 28%, and for wild-type horses was 27%.

## Discussion

Interest in physiological effects associated with the equine *MSTN* promoter SINE mutation has gained traction given its strong association with race distance aptitude^15,16,21^ and with fracture risk in Thoroughbreds^24^. Indeed, genotyping for the linked intronic SNP is promoted as a training and racing aid^21^. Our study confirms the highly significant differences in plasma myostatin concentration between horses of the different *MSTN* genotypes seen by us previously^23^. We similarly confirm the substantial overlap in plasma myostatin concentration in wild type and heterozygous horses and now reveal the relative consistency of this metric within individual horses across different seasons. In particular, within heterozygotes and wild type horses, there are animals with persistently higher or lower plasma myostatin concentrations, evidenced by the relatively low intra-horse CV % (17% and 18%, respectively), when compared to the inter-horse variation (28% and 27%, respectively). This confirms that the variation seen in these genotypes is not entirely as a result of intra-horse variability.

It remains to be seen whether measurement of plasma myostatin might then offer additional insight in racing or exercise performance, muscle phenotype and fracture risk within a defined *MSTN* genotype. Further work in a larger number of horses will be necessary to determine whether this is the case.

Plasma myostatin concentrations had limited fluctuation over the study period, with no seasonal variation, although the limited number of homozygous horses in the study population might have limited the power to detect seasonal variation within that group. The horses in this study were kept under similar management, and samples were obtained at the same time of day following morning exercise training. Collectively, this likely minimised the impact of external and environmental factors to the best of our ability. However, plasma myostatin has been demonstrated to increase following exercise in a group of Arabian horses^25^ and although the horses we studied were on broadly similar training regimes, precise control of exercise intensity, racing schedule and sampling time relative to exercise could not be achieved. We therefore cannot rule out the possibility of a confounding effect of training or detraining and season on circulating myostatin. Consequently, in order to mitigate possible compounding effects of recent haemodynamic changes associated with exercise, relative dehydration or other factors, we determined whether normalising plasma myostatin concentration to plasma albumin might reduce variation in measured myostatin in individual animals but no differences were obtained. This suggests that haemoconcentration did not account for fluctuations in plasma myostatin in our horses.

Further environmental considerations, such as temperature and daylight were also not specifically controlled for, although trends related to these factors would likely be highlighted by the seasonal analysis. No association with age was identified either in this study, or in our previous work^23^, although both studies had a relatively young population of adult horses meaning that older age-related changes might still occur in this species^26^.

Experimental inconsistencies, such as sample handling and storage conditions, as well as assay variability, are likely to have contributed to variability. To account for plate-to-plate variability ELISA plate was included in the model as a random effect, but we cannot exclude some experimental variation related to pipetting errors or other variables. Nonetheless, the observed consistency in myostatin concentrations within horses suggests that any methodological variation was likely low.

While *MSTN* genotype significantly influences circulating myostatin, with the lowest plasma concentrations being obtained consistently in homozygotes, there remains a prominent range of myostatin concentrations with marked overlap, between particularly heterozygotes and wild type horses. The consistency within individual animals, after controlling for management and environmental effects suggests that other factors influence plasma myostatin in horses. Both age and sex were not associated with plasma myostatin; it seems likely then that there are alternative genetic reasons that influence plasma myostatin in individual Thoroughbreds, beyond the *MSTN* SINE mutation. Regulation of *MSTN* expression is complex, occurring at transcriptional, post transcriptional and at epigenetic levels^31^: future study, examining genomic or epigenetic differences with myostatin as a quantitative trait, within SINE-genotyped groups, might help elucidate additional factors that contribute to these differences.

The physiological consequences of the consistency of myostatin seen in our study are unclear. Previous research in mice^32^showed that moderate reductions in myostatin (down to 40% of normal) do not lead to significant muscle hypertrophy; the latter is only seen when myostatin levels are reduced to less than 10% of normal. However, physiological effects of different plasma myostatin might be very different in horses that are bred for their elite exercise performance, and where marginal gains can be the distinction between winning and losing. Future studies could examine performance, muscle characteristics and fracture risk in horses within defined *MSTN* genotypes of either high or low plasma myostatin concentrations. It will also be of interest to determine whether plasma myostatin correlates with muscle *MSTN* gene expression.

In summary, our study highlights the relative consistency of circulating myostatin concentrations within individual Thoroughbreds over time and confirms the major differences between *MSTN* genotyped groups. We reveal that individual horses have relatively higher or lower plasma myostatin concentrations within *SINE* genotyped groups (i.e. low intra-horse variation). The overlap in myostatin concentrations between heterozygous and wild-type horses despite similar management suggests there might be other contributing genetic factors. Further work is required to determine whether circulating myostatin is a useful biomarker for predicting performance and fracture risk.

## Supplementary Information

Below is the link to the electronic supplementary material.

Supplementary Material 1

## Data Availability

The data that support the findings of this study are available from the corresponding author upon reasonable request.
