# Supplementary material for: Temporal and intra-horse consistency of circulating myostatin concentrations in Thoroughbred racehorses
Source: Sci Rep. 2025 Nov 5;15:38708. doi: 10.1038/s41598-025-22472-7 (PMC12589566; doi:10.1038/s41598-025-22472-7)
Supplement: Supplementary file 1 — Supplementary Material 1 [file 41598_2025_22472_MOESM1_ESM.docx]

Supplementary File 1

**Analysis of Variance: Matrix and Storage**

*>stability_anova <- aov(MSTN ~ Sample * Storage, data = Stability_ELISA)*

*>resid_stability <- residuals(stability_anova)*

*>shapiro.test(resid_stability)*

Shapiro-Wilk normality test

data: resid_stability

W = 0.94133, p-value = 0.04212

*>leveneTest(MSTN ~ Sample * Storage, data = Stability_ELISA)*

Levene's Test for Homogeneity of Variance (center = median)

Df F value Pr(>F)

group 7 0.1072 0.9974

31

*>summary( aov(MSTN ~ Sample * Storage + Error(Horse/(Sample*Storage)), data = Stability_ELISA))*

Error: Horse

Df Sum Sq Mean Sq F value Pr(>F)

Sample 1 0.0003 0.0003 0 0.544

Residuals 3 1.7015 0.5672

Error: Horse:Sample

Df Sum Sq Mean Sq F value Pr(>F)

Sample 1 0.006243 0.006243 1.543 0.302

Storage 1 0.006036 0.006036 1.492 0.309

Residuals 3 0.012139 0.004046

Error: Horse:Storage

Df Sum Sq Mean Sq F value Pr(>F)

Storage 3 0.004073 0.001358 0.536 0.667

Sample:Storage 1 0.009202 0.009202 3.635 0.083

Residuals 11 0.027842 0.002531

Error: Horse:Sample:Storage

Df Sum Sq Mean Sq F value Pr(>F)

Sample:Storage 3 0.01404 0.00468 1.096 0.392

Residuals 11 0.04697 0.00427

**Analysis of Variance: Genotyope**

*>Anova_genotype <- aov(formula = mean_MSTN ~ Genotype, data = horse_means)*

*>resid_geno <- residuals(Anova_genotype)*

*>shapiro.test(resid_geno)*

Shapiro-Wilk normality test

data: resid_geno

W = 0.90197, p-value = 0.0006375

*>leveneTest(mean_MSTN ~ Genotype, data = horse_means)*

Levene's Test for Homogeneity of Variance (center = median)

Df F value Pr(>F)

group 2 6.4017 0.003525 **

46

*>oneway_genotype <- oneway.test(mean_MSTN ~ Genotype, data = horse_means, var.equal = FALSE)*

One-way analysis of means (not assuming equal variances)

data: mean_MSTN and Genotype

F = 247.29, num df = 2.000, denom df = 28.136, p-value < 2.2e-16

*>* pairwise.t.test(horse_means$mean_MSTN, horse_means$Genotype, p.adjust.method = "bonferroni", pool.sd = FALSE)

Pairwise comparisons using t tests with non-pooled SD

data: horse_means$mean_MSTN and horse_means$Genotype

Homozygous Heterozygous

Heterozygous < 2e-16 -

Wild type 1.1e-09 0.00013

P value adjustment method: bonferroni

**Chi Squared test: Season genotype distribution**

*genotype_season_table <- table(ELISA_long$Genotype, ELISA_long$Season_sampled)*

*chisq.test(genotype_season_table)*

Pearson's Chi-squared test

data: genotype_season_table

X-squared = 6.854, df = 6, p-value = 0.3346

**Mixed models**

*library(lmerTest)*

*fit1 <- lmer(MSTN_norm ~ Genotype * Season_sampled + (1 | ID) + (1|`ELISA#`), data=ELISA_long)*

*hist(resid(fit1))*

*anova(fit1)*

Type III Analysis of Variance Table with Satterthwaite's method

Sum Sq Mean Sq NumDF DenDF F value Pr(>F)

Genotype 607313 303657 2 51.944 82.0968 <2e-16 ***

Season_sampled 11295 3765 3 197.351 1.0179 0.0838

Genotype:Season 25245 4207 6 195.745 1.1375 0.7720

*Fit2 <- lmer(MSTN_norm ~ Genotype * Sex * Days_between + (1 | ID) + (1|`ELISA#`), data=ELISA_long)*

*hist(resid(fit2))*

*anova(fit2)*

Type III Analysis of Variance Table with Satterthwaite's method

Sum Sq Mean Sq NumDF DenDF F value Pr(>F)

Genotype 195030 97515 2 135.83 25.1830 4.981e-10 ***

Sex 9182 4591 2 143.09 1.1856 0.3085

Days_between 534 534 1 183.21 0.1379 0.7108

Genotype:Sex 11293 2823 4 149.47 0.7291 0.7334

Genotype:Days_b 389 195 2 183.60 0.0503 0.9810

Sex:Days_between 5946 2973 2 183.54 0.7678 0.4655

Genotype:Sex:Days_b 13110 3278 4 184.41 0.8464 0.4974

**ANOVA: CV% Genotype**

*>CV_anova <- aov(COV$COV ~ COV$Genotype)*

*> resid_cv <- (residuals(CV_anova)*

*> shapiro.test(resid_cv)*

*Shapiro-Wilk normality test*

*data: resid_cv*

*W = 0.98409, p-value = 0.7425*

*> leveneTest(COV$COV ~ COV$Genotype)*

Levene's Test for Homogeneity of Variance (center = median)

Df F value Pr(>F)

group 2 0.9173 0.4068

46

*> summary(CV_anova)*

Df Sum Sq Mean Sq F value Pr(>F)

COV$Genotype 2 0.01544 0.007719 1.633 0.207

Residuals 46 0.21750 0.004728

**Student’s t-test: Albumin correction**

*> diffs <- CV_wide$Myostatin - CV_wide$`Albumin-corrected`*

*> shapiro.test(diffs)*

Shapiro-Wilk normality test

data: diffs

W = 0.87342, p-value = 0.1337

*>t.test(CV_wide$Myostatin, CV_wide$`Albumin-corrected`, paired = TRUE)*

Paired t-test

data: CV_wide$Myostatin and CV_wide$`Albumin-corrected`

t = -0.38097, df = 8, p-value = 0.7131

alternative hypothesis: true mean difference is not equal to 0

95 percent confidence interval:

-0.01895299 0.01357857

sample estimates:

mean difference

-0.002687212
